# Japanese Encephalitis Virus NS1′ Protein Interacts with Host CDK1 Protein to Regulate Antiviral Response

**DOI:** 10.1128/Spectrum.01661-21

**Published:** 2021-11-10

**Authors:** Qiuyan Li, Dengyuan Zhou, Fan Jia, Luping Zhang, Usama Ashraf, Yunchuan Li, Hongyu Duan, Yunfeng Song, Huanchun Chen, Shengbo Cao, Jing Ye

**Affiliations:** a State Key Laboratory of Agricultural Microbiology, Huazhong Agricultural University, Wuhan, Hubei, People’s Republic of China; b Laboratory of Animal Virology, College of Veterinary Medicine, Huazhong Agricultural University, Wuhan, Hubei, People’s Republic of China; c The Cooperative Innovation Center for Sustainable Pig Production, Huazhong Agricultural University, Wuhan, Hubei, People’s Republic of China; d Shenzhen Institutes of Advanced Technology, Chinese Academy of Sciences, Shenzhen, Guangdong, People’s Republic of China; Regional Centre for Biotechnology

**Keywords:** Japanese encephalitis virus, NS1′, CDK1, CREB, c-Rel

## Abstract

Type I interferon (IFN-I) is a key component of the host innate immune system. To establish efficient replication, viruses have developed several strategies to escape from the host IFN response. Japanese encephalitis virus (JEV) NS1′, a larger NS1-related protein, is known to inhibit the mitochondrial antiviral signaling (MAVS)-mediated IFN-β induction by increasing the binding of transcription factors (CREB and c-Rel) to the microRNA 22 (miRNA-22) promoter. However, the mechanism by which NS1′ induces the recruitment of CREB and c-Rel onto the miRNA-22 promoter is unknown. Here, we found that JEV NS1′ protein interacts with the host cyclin-dependent kinase 1 (CDK1) protein. Mechanistically, NS1′ interrupts the CDC25C phosphatase-mediated dephosphorylation of CDK1, which prolongs the phosphorylation status of CDK1 and leads to the inhibition of MAVS-mediated IFN-β induction. Furthermore, the CREB phosphorylation and c-Rel activation through the IκBα phosphorylation were observed to be enhanced upon the augmentation of CDK1 phosphorylation by NS1′. The abrogation of CDK1 activity by a small-molecule inhibitor significantly suppressed the JEV replication *in vitro* and *in vivo*. Moreover, the administration of CDK1 inhibitor protected the wild-type mice from JEV-induced lethality but showed no effect on the MAVS^–/–^ mice challenged with JEV. In conclusion, our study provides new insight into the mechanism of JEV immune evasion, which may lead to the development of novel therapeutic options to treat JEV infection.

**IMPORTANCE** Japanese encephalitis virus (JEV) is the main cause of acute human encephalitis in Asia. The unavailability of specific treatment for Japanese encephalitis demands a better understanding of the basic cellular mechanisms that contribute to the onset of disease. The present study identifies a novel interaction between the JEV NS1′ protein and the cellular CDK1 protein, which facilitates the JEV replication by dampening the cellular antiviral response. This study sheds light on a novel mechanism of JEV replication, and thus our findings could be employed for developing new therapies against JEV infection.

## INTRODUCTION

Japanese encephalitis virus (JEV), which is transmitted by mosquitoes, is one of the primary causes of acute viral encephalitis in humans in Asia. It is a typical neurotropic virus belonging to the family *Flaviviridae*, which includes many serious human pathogens, such as Zika virus (ZIKV), dengue virus (DENV), yellow fever virus, and West Nile virus (1, 2). Despite having effective vaccines, but no specific treatment, JEV causes more than 50,000 cases per year, with a case fatality rate of 20% to 30%. Among the survivors, 30% to 50% develop permanent neurological sequelae (3, 4). JEV is a single-stranded, positive-sense RNA virus with one open reading frame which encodes three structural proteins (C, prM, and E) and seven nonstructural proteins (NS1, NS2A, NS2B, NS3, NS4A, NS4B, and NS5) (2). In addition to these 10 proteins, viruses from the JEV serogroup produce a unique NS1-related protein, designated NS1′, which is the product of a −1 ribosomal frameshift and consists of the entire NS1 with a 52-amino acid (aa) C-terminal extension (the first 9 aa of NS2A protein and an additional 43-aa stretch) (5–7). Like many viruses, multiple immune evasion strategies are governed by flaviviruses. For example, NS5 protein induces STAT2 degradation during DENV or ZIKV infection (8, 9). The JEV NS5 protein not only blocks the nuclear translocation of IRF3 and NF-κB (10) but also inhibits the TYK2 phosphorylation (11). The West Nile virus NS1 protein reduces interferon β (IFN-β) production by interacting with and promoting the degradation of retinoic acid-inducible gene 1 (RIG-I) and melanoma differentiation-associated protein (MDA5) (12). Studies with duck Tembusu virus, an emerging flavivirus of ducks, showed that NS1 interacts with MAVS to inhibit the IFN-β activation (13). Similarly, the JEV NS1′ protein has also been shown to antagonize the MAVS-mediated IFN-I production by enhancing the miR-22 expression in our previous study (14). However, how the JEV NS1′ protein promotes the activation of transcription factors (TFs) to enhance the miR-22 expression is still unclear.

Cyclin-dependent kinases (CDKs) are a family of serine/threonine kinases whose sequential activation and inactivation ensure the unidirectional progression of the cell cycle (15). CDK1 is a crucial determinant of mitotic progression, which together with the cyclin B coordinates entry to mitosis (16). The enzymatic activity of cyclin B1-CDK1 depends on the phosphorylation state of CDK1: a conserved threonine residue in the activation loop (Thr-161 in human) must be phosphorylated and two sites in the ATP-binding pocket (Thr-14 and Tyr-15 in human) must be dephosphorylated (17). Kinases of the Wee1/Myt1 family phosphorylate Thr-14 and Tyr-15, whereas phosphatases of the CDC25C family dephosphorylate both of these sites (17). Due to its role in cell cycle regulation, CDK1 is considered a potential target for cancer therapy (18–22). Moreover, CDK1 is known to be involved in the regulation of viral infections. The human parvovirus B19 NS1 protein induces G_2_-phase arrest by inactivating the cyclin B-CDK1 complex (23). The avian reovirus p17 protein inhibits the CDK1-mediated vimentin phosphorylation, which causes cell cycle arrest and facilitates virus replication (24). CDK1 also regulates antiviral immunity against the human immunodeficiency virus 1 infection by maintaining the phosphorylation of the antiviral factor SAMHD1 (25). Whether or not CDK1 is involved in regulating the JEV infection remains unknown.

In this study, CDK1 protein was identified to interact with JEV NS1′ protein. Further results showed that NS1′ interrupts the CDC25C phosphatase-mediated dephosphorylation of CDK1 to augment the phosphor-CDK1, which suppresses the induction of MAVS by increasing the binding of CREB and c-Rel to the promoter region of miR-22. These findings reveal a novel mechanism of NS1′-mediated immune evasion and highlight therapeutic targets to control JEV infection.

## RESULTS

### JEV NS1′ protein interacts with the host CDK1 protein.

**FIG 1:**
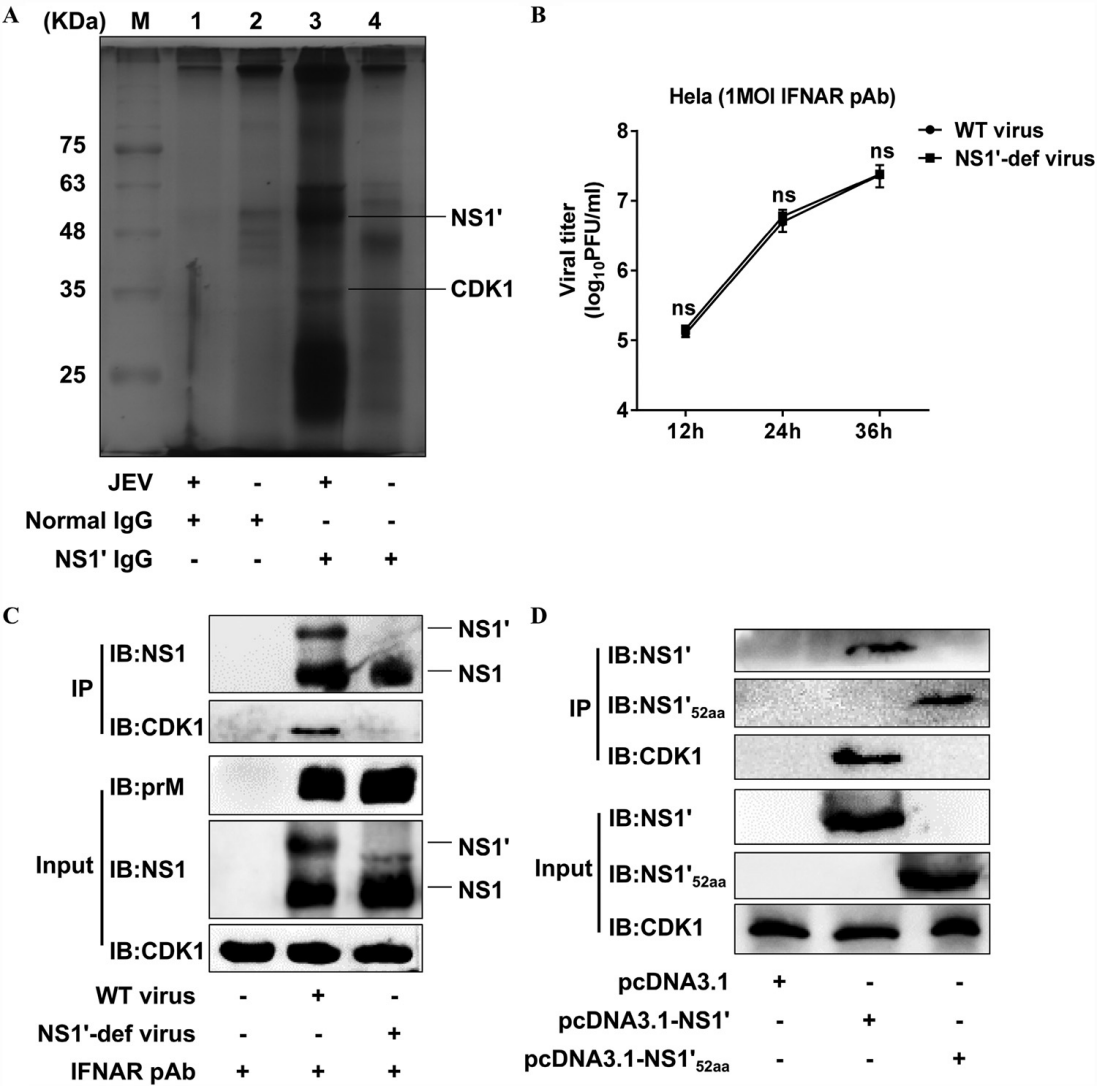
Identification of interaction between JEV NS1′ and host CDK1 protein. (A) HeLa cells were infected with WT JEV at an MOI of 1.0 for 36 h followed by the co-IP analysis of cell lysates with JEV NS1′ antibodies. The purified proteins were visualized by silver staining. The protein bands in lane 3 which are different from those in lane 4 were excised and analyzed using the LC-MS/MS. The position and names of proteins identified by MS are indicated. (B) HeLa cells treated with IFNAR polyclonal antibodies (pAb) were mock-infected or infected with WT virus or NS1′-def virus at an MOI of 1.0, and the virus titers in the culture supernatants were measured by plaque assay at 12, 24, and 36 hpi. (C) HeLa cells were treated with IFNAR pAb and then infected with WT virus or NS1′-def virus at an MOI of 1.0 for 36 h, followed by co-IP analysis of cell lysates with JEV NS1 antibodies. Cellular proteins coimmunoprecipitated by respective antibodies were analyzed by Western blotting. (D) HeLa cells were transfected with pcDNA3.1 plasmids expressing the NS1′, ΔNS1′_52aa_, or empty vector for 36 h, followed by co-IP analysis of cell lysates with JEV ΔNS1′_52aa_ antibodies. Cellular proteins coimmunoprecipitated by respective antibodies were analyzed by Western blotting. All data in panels B to D were pooled from three independent experiments. ns, nonsignificant.

We previously found that JEV NS1′ protein inhibits the miR-22-MAVS-mediated IFN-β induction by increasing the binding of CREB and c-Rel to the miR-22 promoter (14). To explore the potential mechanism by which JEV NS1′ protein recruits CREB and c-Rel onto the miR-22 promoter, we screened the proteins interacting with NS1′ from JEV-infected cells using the NS1′-specific monoclonal antibody. The amino acid sequence identity of the visualized discrepant protein bands between JEV- and mock-infected cells revealed that several host proteins, including CDK1, are likely to interact with NS1′ (Fig. 1A). To confirm whether the observed findings are specific to NS1′, a recombinant NS1′-deficient JEV virus (NS1′-def virus) was used, which replicates equivalent to the wild-type JEV virus (WT virus) in the presence of IFN-α/β receptor (IFNAR) polyclonal antibodies (26) (Fig. 1B). The interactions of NS1′ with CDK1 were further examined by coimmunoprecipitation (co-IP) assay, and the results showed that during infection with the WT virus, but not with the NS1′-def virus, CDK1 was coprecipitated with NS1′ protein (Fig. 1C). In order to delineate the NS1′ protein domain interacting with CDK1, cultured cells were transfected with plasmids expressing the full-length NS1′, ΔNS1′_52aa_ (the last 52 aa of NS1′ protein), or empty vector, and subsequently, the cell lysates were subjected to co-IP analysis. Compared to the interaction between the full-length NS1′ and CDK1, no interaction between ΔNS1′_52aa_ and CDK1 was detected (Fig. 1D). Altogether, these results suggest that JEV NS1′ interacts with CDK1, which relied on the overall structure of NS1′ protein rather than the ΔNS1′_52aa_.

### JEV NS1′ promotes the CDK1 phosphorylation by CDC25C.

**FIG 2:**
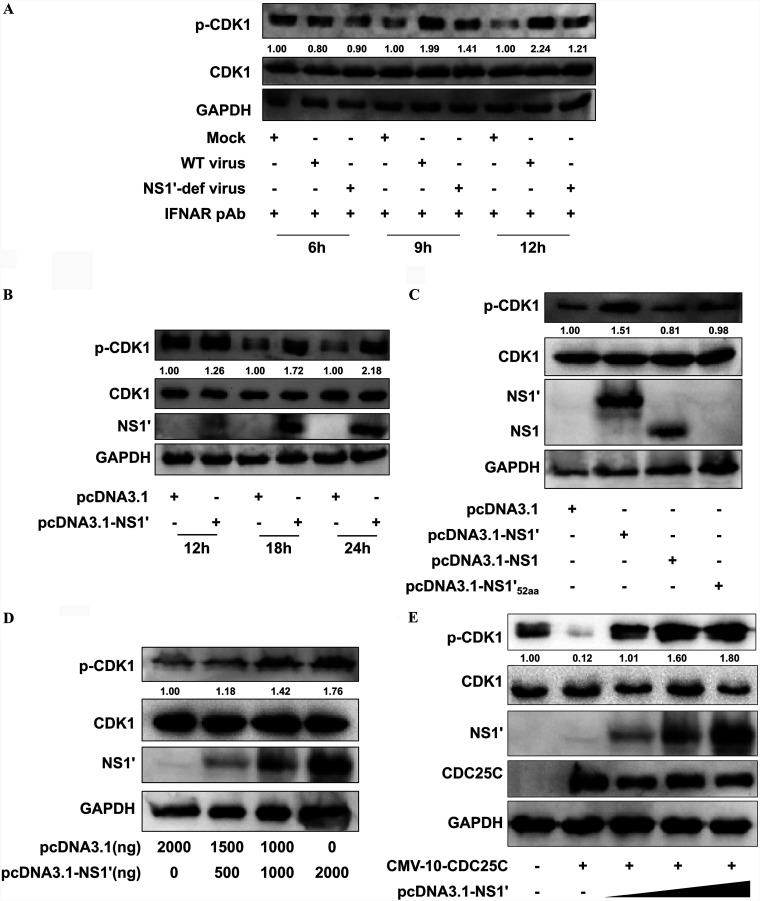
JEV NS1′ enhances the CDK1 phosphorylation by reducing the CDC25C function. (A) HeLa cells treated with IFNAR pAb were mock-infected or infected with WT virus or NS1′-def virus at an MOI of 1.0, and then the cells were harvested at 6, 9, and 12 hpi. Whole-cell protein lysates were separated by SDS-PAGE and examined by Western blotting using indicated antibodies. (B) HeLa cells were transfected with pcDNA3.1-NS1′ or empty vector for 12, 18, and 24 h, and cell lysates were subjected to Western blotting using indicated antibodies. (C) HeLa cells were transfected with pcDNA3.1 plasmids encoding the JEV NS1, NS1′, or ΔNS1′_52aa_, or empty vector for a period of 18 h. Total cell lysates were collected for analysis by Western blotting with indicated antibodies. (D) HeLa cells were cotransfected with empty vector and an increasing amount (0, 500, 1,000, or 2,000 ng) of pcDNA3.1-NS1′. The expression of p-CDK1, CDK1, and JEV NS1′ protein was detected by Western blotting with indicated antibodies. (E) HeLa cells were cotransfected with p3×FLAG-CMV-10-CDC25C and different dosages of pcDNA3.1-NS1′. Cells were harvested at 24 h posttransfection and were subjected to Western blotting with indicated antibodies. Protein levels of p-CDK1 were quantified by immunoblot scanning using image J software and normalized to the amount of GAPDH.

To determine the effect of NS1′-CDK1 interaction on the CDK1 activity, the CDK1 expression and phosphorylation at the Tyr-15 residue were examined in cultured cells infected with WT or NS1′-def viruses. Under conditions which permit both WT and NS1′-def viruses to replicate similarly (Fig. 1B), no obvious alteration of the CDK1 expression was detected upon infection with WT or NS1′-def viruses (Fig. 2A). However, the WT virus showed phosphorylation of CDK1 at the Tyr-15 enhanced compared to that of the mock or NS1′-def virus (Fig. 2A). These results demonstrated that the increasing of CDK1 phosphorylation, but not its expression, was caused mainly by NS1′ protein during JEV infection. To further verify whether JEV NS1′ could directly modulate the CDK1 phosphorylation, cells were transfected with plasmids expressing the JEV NS1′, NS1, ΔNS1′_52aa_, or empty vector. Compared with empty vector, NS1, or ΔNS1′_52aa_, a prominent increase in the CDK1 phosphorylation at the Tyr-15 was observed upon overexpression of NS1′ (Fig. 2B and C), which maintained in the dose-dependent manner (Fig. 2D), suggesting that NS1′ plays an important role in enhancing the CDK1 phosphorylation at the Tyr-15 during JEV infection.

Given that CDK1 activity varies with the cell cycle, cells were synchronized by serum starvation prior to detection of the effect of NS1′ on CDK1 phosphorylation. Similar to the results from normal cells, the WT virus enhanced phosphorylation of CDK1 at the Tyr-15 compared to the mock or NS1′-def virus (Fig. S1A). Also, compared with empty vector, NS1′-expresing plasmid can prominently increase the levels of phosphorylated CDK1 (Fig. S1B).

It is known that CDC25C activates CDK1 by dephosphorylating at the tyrosine residue 15 site (27). Given that the CDK1 phosphorylation at the Tyr-15 is enhanced upon JEV NS1′ expression, we asked whether NS1′ blocks the CDC25C-mediated dephosphorylation of CDK1. To this end, the increasing amount of plasmid expressing the NS1′ was cotransfected with the CDC25C-expressing plasmids in cultured cells. It was observed that NS1′ inhibited CDC25C-mediated dephosphorylation of CDK1 in a dose-dependent manner, which resulted in augmentation of phosphorylated CDK1 at the Tyr-15 (Fig. 2E). Overall, these data suggest that JEV NS1′ promotes the CDK1 phosphorylation at the Tyr-15, which may be associated with the reduced activity of CDC25C.

### Phosphorylated CDK1 positively regulates the replication of JEV.

**FIG 3:**
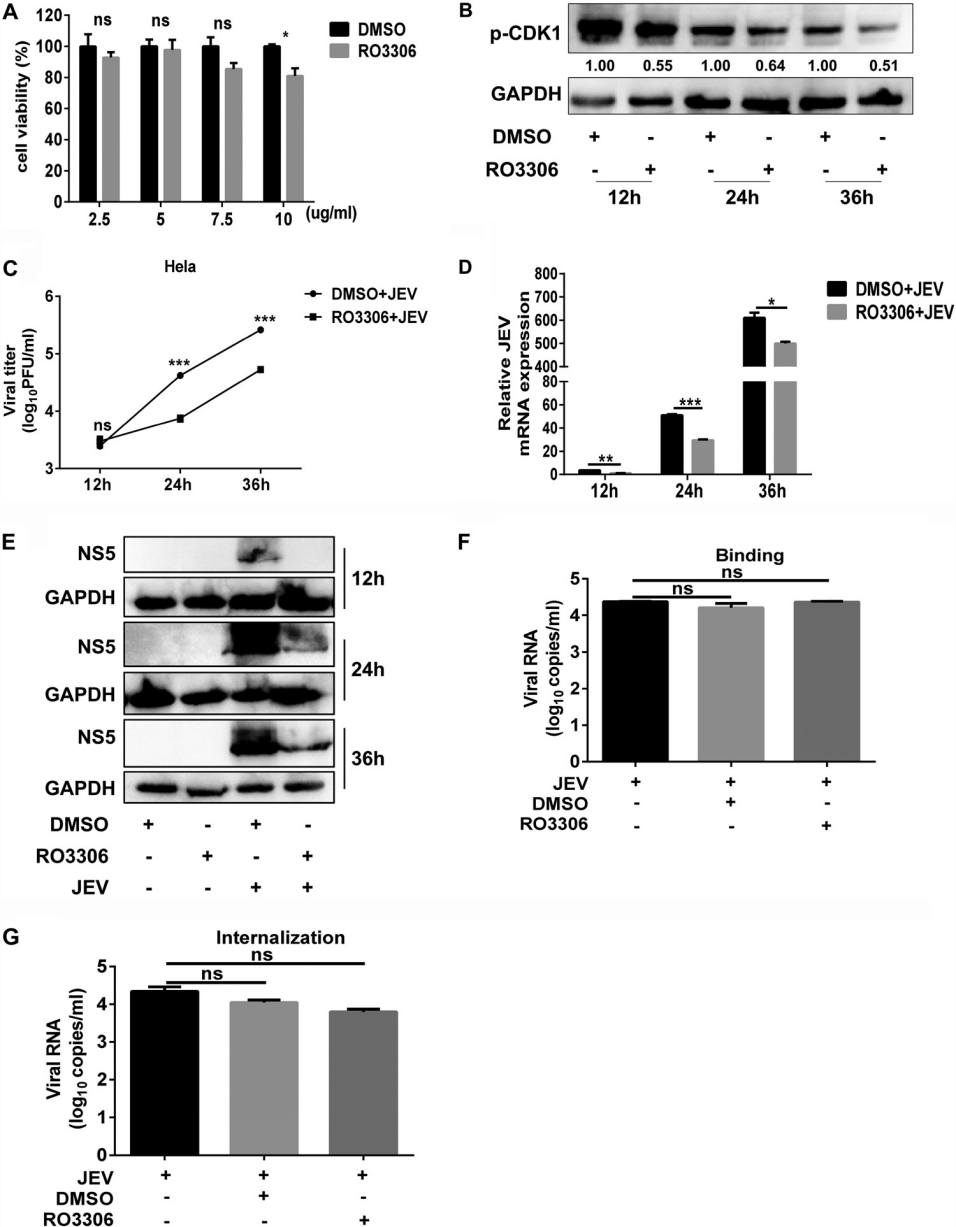
CDK1 positively regulates the replication of JEV. (A) HeLa cells viability assay at indicated concentrations of RO3306 or DMSO. (B) HeLa cells were treated with 5 μg/ml RO3306 or DMSO for 12, 24, and 36 h. Total cell lysates were collected for analysis by Western blotting with indicated antibodies. (C to E) HeLa cells were treated with 5 μg/ml RO3306 or DMSO for 2 h, and then cells were mock infected or infected with the WT virus at an MOI of 1. At 12, 24, and 36 hpi, viral titers (C), total viral mRNA expression (D), and JEV NS5 protein expression (E) were measured by plaque assay, Western blotting, and qRT-PCR, respectively. (F and G) HeLa cells were treated with 5 μg/ml RO3306 or DMSO for 2 h, and then cells were mock infected or infected with the WT virus at an MOI of 10 to examine the virus-cell binding (F) and virus entry into the cells (G) as mentioned in Materials and Methods. Protein levels of p-CDK1 were quantified by immunoblot scanning using image J software and normalized to the amount of GAPDH. All data were pooled from three independent experiments. *, *P* < 0.05; **, *P* < 0.01; ***, *P* < 0.001. ns, nonsignificant.

To determine the impact of CDK1 on JEV replication, a well-characterized small-molecule compound, RO3306, which competitively inhibits the phosphorylation of CDK1 at the Tyr-15, was used. First, the effects of RO3306 on cell viability and phosphorylation of CDK1 were determined. As shown in Figure 3, RO3306 showed no impact on cell viability at high concentration (7.5 μg/ml) (Fig. 3A), and decreased phosphorylation levels at the Tyr-15 of CDK1 protein were observed at noncytotoxic concentration (Fig. 3B). Viral replication was subsequently examined upon treatment of RO3306 or dimethyl sulfoxide (DMSO). It was found that RO3306 inhibited the production of viral infectious particles and the expression of viral NS5 protein and total mRNAs at noncytotoxic concentration (Fig. 3C to E). To exclude the function of CDK1 on cell cycle, the impact of CDK1 on JEV replication was analyzed in cells synchronized by serum starvation. Consistently, RO3306 inhibited the production of viral infectious particles and the expression of viral NS5 protein and total mRNAs at noncytotoxic concentration (Fig. S2A to C). These results indicate that CDK1 plays a positive regulatory role during JEV replication, and this effect is independent on its function on cell cycle.

Considering that CDK1 may affect the phosphorylation status of some cell surface proteins (28), there is a possibility that the suppression of JEV replication by RO3306 may have occurred through impeding the virus entry into the cells. To study this, we evaluated viral binding to the cell surface and internalization in the presence or absence of RO3306 as described in Materials and Methods. No significant differences were noticed in the accumulation of viral RNAs in infected cells subjected to viral binding and internalization assays upon treatment with or without RO3306 (Fig. 3F and G). Hence, these data indicate that phosphor-CDK1 acts as a positive regulator of JEV replication.

### JEV NS1′ inhibits the miR-22-MAVS-mediated IFN-β response via CDK1.

**FIG 4:**
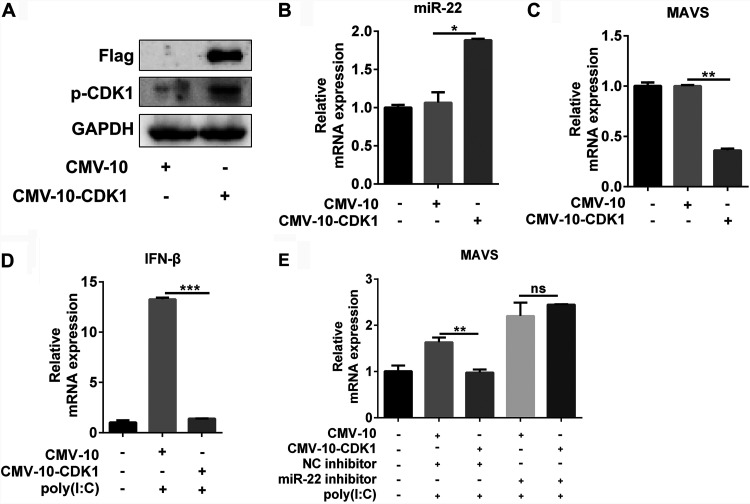
CDK1 inhibits the MAVS-mediated IFN-β induction by increasing the miR-22 expression. (A to C) HeLa cells were transfected with 3×FLAG-CMV-10-CDK1 or an empty vector for 24 h. Samples were collected and were processed for Western blotting to detect the phosphorylation status of CDK1 protein (A) and for qRT-PCR analysis to determine the mRNA levels of miR-22 (B) and MAVS (C). (D) HeLa cells were transfected with 3×FLAG-CMV-10-CDK1 or an empty vector for 24 h. Subsequently, cells were subjected to poly(I·C) treatment for a period of 12 h, and then the mRNA levels of IFN-β were measured by qRT-PCR. (E) HeLa cells were cotransfected with 3×FLAG-CMV-10-CDK1 or empty vector and miR-22 inhibitor or negative-control (NC) inhibitor (final concentration, 100 nM) for 24 h, followed by treatment of cells with poly(I·C) for another 12 h. qRT-PCR was performed to analyze the mRNA levels of MAVS. All data were pooled from three independent experiments. *, *P* < 0.05; **, *P* < 0.01; ***, *P* < 0.001. ns, nonsignificant.

In the previous study, we showed that NS1′ inhibits the MAVS-mediated IFN-β induction by increasing the miR-22 expression (14). To investigate whether CDK1 phosphorylation is involved in regulating the miR-22-mediated IFN-β response, we first examined the expression of miR-22, MAVS, and IFN-β in cells transfected with FLAG-tagged CDK1 plasmid or empty vector. Increased synthesis and phosphorylation at the Tyr-15 levels of CDK1 protein were observed in cells overexpressing the CDK1 (Fig. 4A). Further experiments revealed that the overexpression of CDK1 in cells significantly enhanced the miR-22 expression and suppressed the MAVS and IFN-β expression (Fig. 4B to D). In contrast, the coexpression of CDK1 with miR-22-specific inhibitors in cultured cells impaired the CDK1-induced suppression of MAVS expression (Fig. 4E).

**FIG 5:**
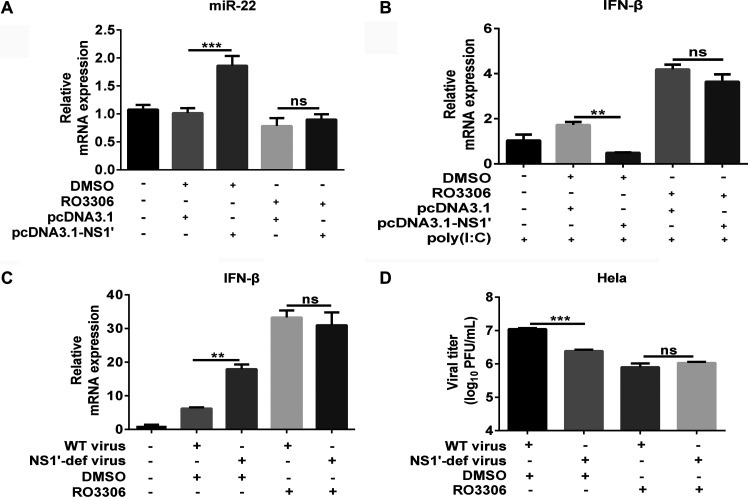
JEV NS1′ inhibits the miR-22-mediated IFN-β response via CDK1. (A) HeLa cells were transfected with pcDNA3.1 plasmids encoding the JEV NS1′ protein or an empty vector. After 24 h transfection, the cells were treated with 5 μg/ml RO3306 or DMSO for a period of 12 h. The mRNA level of miR-22 was analyzed by qRT-PCR. (B) HeLa cells were subjected to transfection and RO3306 treatment paradigm as described in panel A, followed by poly(I·C) treatment for 12 h. The mRNA level of IFN-β was analyzed by qRT-PCR. (C and D) HeLa cells treated with 5 μg/ml RO3306 or DMSO for 2 h were infected with the WT virus or NS1′-def virus at an MOI of 1.0. At 24 hpi, the mRNA level of IFN-β was determined by qRT-PCR (C) and the viral titer in the supernatant was measured by plaque assay (D). All data were pooled from three independent experiments. **, *P* < 0.01; ***, *P* < 0.001. ns, nonsignificant.

We next questioned whether JEV NS1′ inhibits the miR-22-mediated IFN-β response through CDK1. To this end, cultured cells treated with CDK1 inhibitor RO3306 or DMSO were subjected to transfection with plasmids expressing the NS1′ or empty vector. As expected, the NS1′-induced upregulation of miR-22 and suppression of IFN-β were found to be impaired by RO3306 treatment compared to DMSO treatment (control) (Fig. 5A and B), suggesting that the inhibition of CDK1 activity could significantly damage the NS1′ function in inhibiting the miR-22-mediated IFN-β response. To verify these findings during JEV infection conditions, the cells were treated with or without RO3306, followed by the WT virus or NS1′-def virus infection. The RO3306 treatment impaired the WT virus-induced reduction of IFN-β expression (Fig. 5C), and the difference of virus replication in RO3306-treated or untreated cells was also abrogated upon inhibition of the CDK1 activity (Fig. 5D). Taken together, these findings demonstrate that CDK1 is responsible for the NS1′-mediated suppression of IFN-I during JEV infection.

### Phosphorylated CDK1 increases the activation of CREB and c-Rel for their binding to the miR-22 promoter.

**FIG 6:**
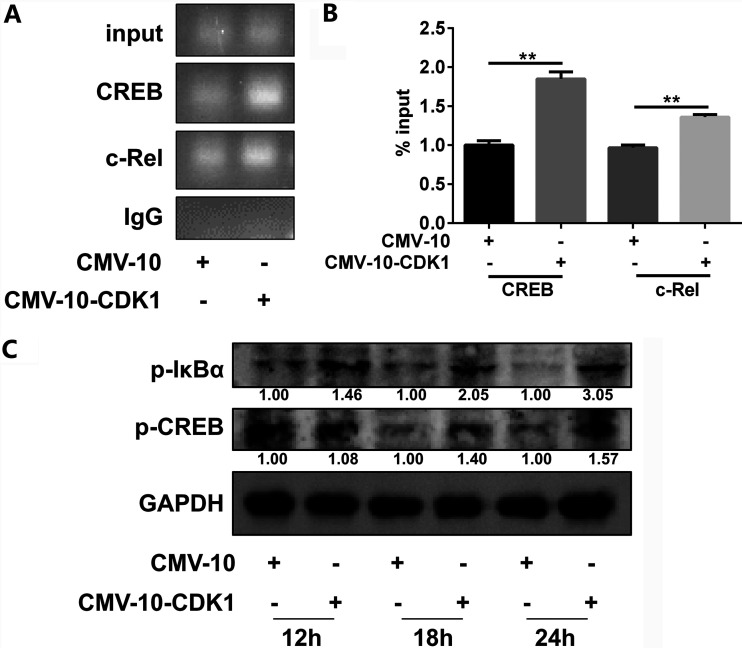
CDK1 increases the CREB and c-Rel activation and enhances their binding to the miR-22 promoter. (A and B) HeLa cells were transfected with 3×FLAG-CMV-10-CDK1 or empty vector for 24 h. Fixed chromatin samples were prepared and immunoprecipitated by using antibodies specific to c-Rel and CREB or by using nonimmune IgG. The enrichment of the c-Rel- and CREB-binding site onto the miR-22 promoter in the ChIP product was analyzed by PCR. The PCR products were separated by acrylamide gel electrophoresis. Representative gels are shown in panel A, and densitometry analysis is presented in panel B. (C) HeLa cells were transfected with 3×FLAG-CMV-10-CDK1 or empty vector for 12, 18, and 24 h. Cell lysates were analyzed by Western blotting with indicated antibodies. Protein levels of p-IκBα and p-CREB were quantified by immunoblot scanning using image J software and normalized to the amount of GAPDH. **, *P* < 0.01.

Given that NS1′ upregulates miR-22 by enhancing the binding of transcription factors (TFs), CREB and c-Rel, to its promoter, we sought to identify the effects of CDK1 on the binding of CREB and c-Rel to the miR-22 promoter. As assessed by the chromatin immunoprecipitation (ChIP) assay, the ectopic expression of CDK1 in cultured cells showed an increase in the binding of c-Rel and CREB to the miR-22 promoter compared with that in the cells transfected with empty vector (Fig. 6A and B). Some studies have demonstrated that CDK1 regulates the activation of TFs, such as Foxo1, by changing their phosphorylation status (29, 30). Considering that the phosphorylation of CREB at Ser-133 stimulates its transcriptional activity (31), the phosphorylation levels of CREB were determined in CDK1-expressing cells. The results showed that the CDK1 overexpression markedly enhanced the CREB phosphorylation at Ser-133 (Fig. 6C). In contrast to CREB, the transcriptional activity of c-Rel depends on the phosphorylation of IκBα at Ser-36 which controls the IκBα-c-Rel complex shuttling from the cytoplasm to the nucleus (32). Next, the phosphorylation levels of IκBα at Ser-36 in CDK1-expressing or controlled cells were assessed, which revealed increased IκBα phosphorylation upon the expression of CDK1 in transfected cells (Fig. 6C). These data indicate that CDK1 regulates the activation of CREB and c-Rel by phosphorylating them in order to promote their binding to the miR-22 promoter.

### CDK1 inhibitor treatment reduces lethality in JEV-infected WT mice but not in MAV_S_^−/−^ mice.

**FIG 7:**
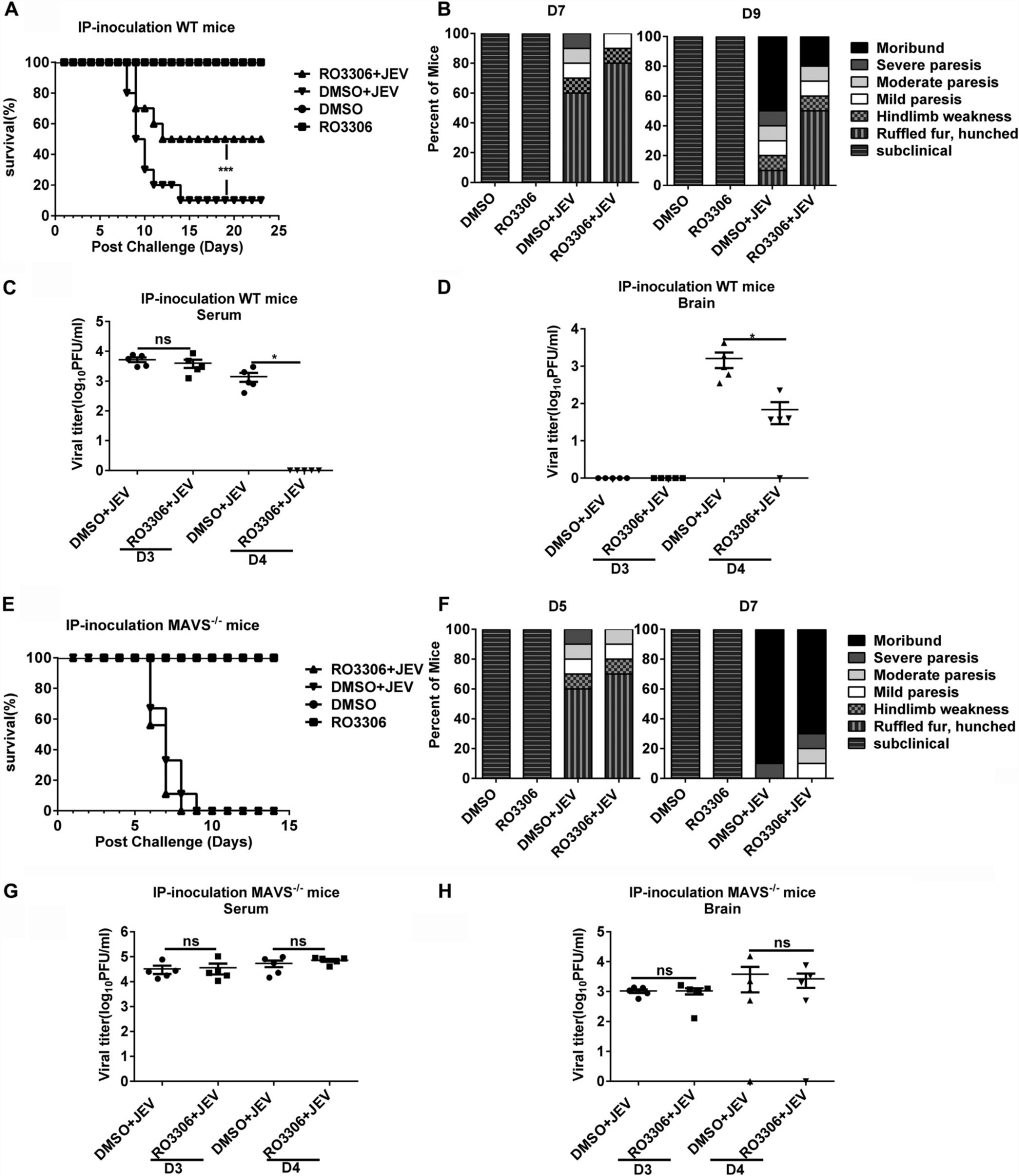
RO3306 treatment reduces lethality in JEV-infected WT mice. (A to H) WT or MAVS^−/−^ mice were infected intraperitoneally with 1 × 10 ^4^ PFU of the JEV virus or the same volume of DMEM. RO3306 (10 mg/kg body weight) or an equal volume of DMSO was intravenously administered on day 3 postinfection. Mice were sacrificed on days 3 (before intravenously administered RO3306) and 4 postinfection, and serum and brain samples were collected for the subsequent analyses. Mice survival rate (A and E) and clinical signs of the disease (B and F) in each experimental group were evaluated for 21 days postinfection (*n* = 10). Viral titers in serum (C and G) and brain samples (D and H), harvested on days 3 and 4 postinfection, were measured by plaque assay (*n* = 5). All data were pooled from three independent experiments. *, *P* < 0.05; ***, *P* < 0.001. ns, nonsignificant.

To characterize the role of CDK1 during JEV infection *in vivo*, a WT mouse model of JEV infection was established. The CDK1 activity inhibitor RO3306 or the negative-control DMSO was injected via intravenous route into mice at 3 days after infection with the WT virus. Treatment of the WT virus-infected mice with RO3306 reduced mortality and ameliorated clinical signs of acute encephalitis compared to treatment of the mice with DMSO (Fig. 7A and B). Moreover, the serum samples and brain tissues collected from the virus-infected WT mice at 24 h after the RO3306 treatment displayed viral titers lower than those collected from the DMSO-treated mice (Fig. 7C and D). To further verify that the CDK1-mediated suppression of MAVS is a major contributor to induce the lethality in the WT virus-infected mice, a MAVS^−/−^ mouse model (5-week-old) was used. No significant differences in mortality, clinical signs, and viral titers in serum and brain samples were observed between the RO3306- and DMSO-treated MAVS^−/−^ mice (Fig. 7E to H). These data collectively suggest that the inhibition of CDK1 activity *in vivo* reduces the lethality of the JEV-infected WT mice by enhancing the MAVS expression.

## DISCUSSION

**FIG 8:**
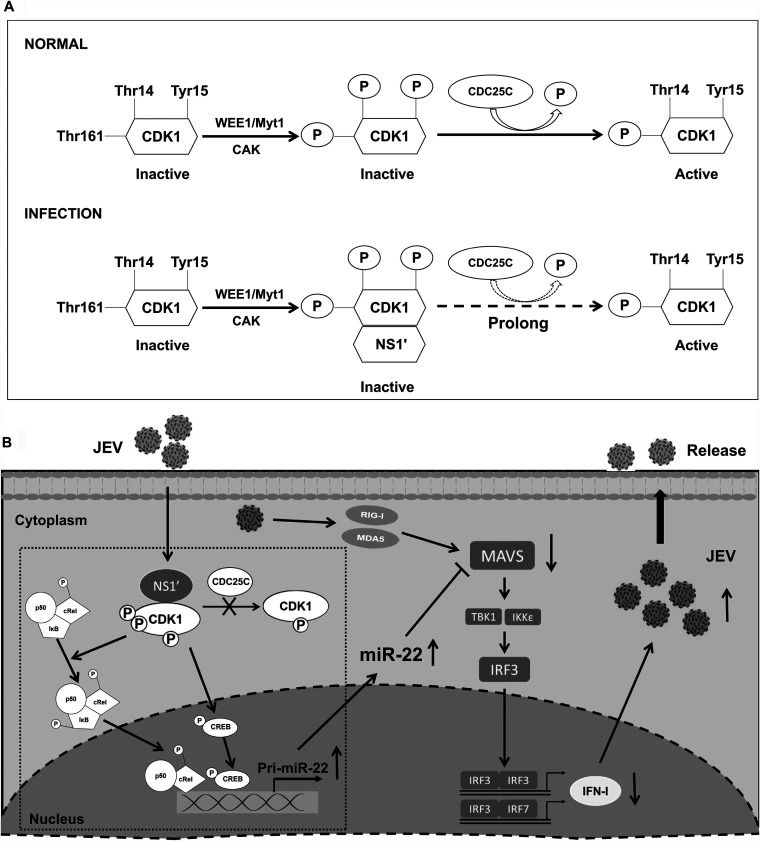
Hypothetical model for the mechanism of JEV NS1′-mediated activation of CREB and c-Rel. (A) The effect of JEV NS1′ on CDK1 phosphorylation status: under the normal condition, a conserved threonine residue in the activation loop (Thr-161) of CDK1 is phosphorylated by CAK, and two sites in the ATP-binding pocket (Thr-14 and Tyr-15) are dephosphorylated. Both of these sites of CDK1 are then phosphorylated by kinases of the Wee1/Myt1 family, and subsequently dephosphorylated by phosphatases of the CDC25C, during JEV infection, and JEV NS1′ interacts with CDK1 and interrupts the CDC25C phosphatase-mediated dephosphorylation of CDK1, which prolongs the phosphorylation status of CDK1. (B) Mechanism of JEV NS1′-mediated activation of CREB and c-Rel. NS1′ protein interacts with CDK1 in host cells to prolong the phosphorylation status of CDK1, which promotes the activation of CREB and c-Rel and leads to increased expression of miR-22. miR-22 reduces the IFN-I production by targeting MAVS. The schematic in the dotted box represents the mechanism demonstrated in the present study, and the other part indicates the mechanism that has been clarified in our previous study (14).

In this study, we demonstrated that JEV NS1′ interacts with CDK1 and inhibits CDC25C-mediated dephosphorylation of CDK1 (Fig. 8A), which enables the activation of CREB and c-Rel for their binding to the prompter elements of miR-22 in order to dampen the MAVS-mediated IFN-I expression (Fig. 8B). These findings elucidate the mechanism for JEV NS1′-mediated activation of CREB and c-Rel, which filled the gap in our previous study (Fig. 8B) (14).

Given that JEV NS1′, but not NS1, interacts with CDK1, we speculated that the last 52 aa of NS1′ (ΔNS1′_52aa_) could be the main region for its interactions with CDK1. Unexpectedly, no interaction between ΔNS1′_52aa_ and CDK1 was detected, suggesting that the NS1′-CDK1 interaction may rely on the unique structure of NS1′ protein. Therefore, resolving the protein structure of NS1′ may provide new insights into the understanding of its interactions with CDK1.

Viruses have evolved several powerful strategies to facilitate their replication effectively, and one of them is the induction of cell cycle arrest (33). For instance, the human papillomavirus E6 protein abrogates cell cycle checkpoints by upregulating CDK1, which successfully induces polyploidy (34). The expression of reovirus nonstructural protein sigma1s causes G_2_/M arrest via inducing the hyperphosphorylation of CDK1 (35). Similarly, the infection of human herpesviruses is known to induce cell cycle arrest by enhancing the CDK1 phosphorylation either through perturbing the activities of Wee1 and CDC25C phosphatase or through inactivating the cyclin B-CDK1 complex (36, 37). In addition, we found that the NS1′-def virus also slightly increases the phosphorylation of CDK1 but to a markedly lesser extent than WT virus, suggesting that some other viral components may also contribute to regulating CDK1 phosphorylation; however, the effect is much weaker than that of NS1′.

To substantiate the role of CDK1 during JEV infection, a commercially available small-molecule compound RO3306 that functionally blocks the CDK1 phosphorylation at the Tyr-15 by acting as an ATP-competitive inhibitor (38) was employed in this study. The RO3306 treatment ameliorated the suppression of NS1′-mediated IFN-β response in cultured cells, which in turn led to the inhibition of JEV replication in cultured cells and WT mice. In addition to RO3306, the inhibitors of some other members of the CDK family have shown promising effects in treating infections caused by flaviviruses. A drug-repurposing approach identified 10 structurally unrelated small-molecule inhibitors of CDKs that exhibited anti-ZIKV properties in human neural cells (39). Another recent high-throughput drug screen demonstrated the use of CDK1, CDK2, and CDK9 inhibitors in restricting the replication of JEV, DENV, and ZIKV in human liver cells (40). Hence, the CDKs other than CDK1 may also be involved in the regulation of JEV replication, and their targeting may offer new anti-JEV therapeutic options.

Instead of an elevated expression level of c-Rel, the translocation of c-Rel from the cytoplasm to the nucleus has been observed to increase in JEV-infected cells (41), suggesting that CDK1 may regulate the nuclear translocation of c-Rel. It is well known that the translocation activity of c-Rel, which partners with IκBα to shuttle from the cytoplasm to the nucleus, depends mainly on the IκBα phosphorylation at Ser-36 (32). Therefore, the phosphorylation of IκBα was examined in CDK1-transfected cells. As expected, the phosphorylation of IκBα was observed to be enhanced upon CDK1 overexpression, indicating that there is a correlation between CDK1 and IκBα-c-Rel complex. However, whether the correlation is direct or through an indirect network requires further investigations. Furthermore, the phosphorylation of CREB at Ser-133, which mainly stimulates its transcriptional activity, increases upon JEV infection (41). Given that CDK1 can regulate the activity of some TFs, such as FOXO1 (30), FoxM1 (42), and TFCP2L1 (43), by phosphorylating them, the phosphorylation of CREB was checked in CDK1-transfected cells. Consistently, we found that the phosphorylation of CREB was upregulated in cells overexpressing the CDK1. Studies have also shown that CDK1 can affect multiple cellular signaling pathways, including apoptosis, by regulating the JAK/STAT3 activity (44). In this study, we focused on the role of CDK1 in regulating the NS1′-mediated suppression of IFN-I production during JEV infection. Determining whether or not CDK1 protein participates in the regulation of apoptosis during JEV infection of neuronal cells would be interesting and may uncover new mechanisms of JEV-induced neuronal cell damage.

In summary, our study demonstrated that JEV NS1′ protein interacts with the host CDK1 to enhance its phosphorylation status, which subsequently promotes the binding of CREB and c-Rel to the miR-22 promoter in order to diminish the MAVS-mediated IFN-I production during JEV infection. Our findings provide novel clues for JEV to evade the host innate immune response which could be considered to establish new therapies against JEV infection.

## MATERIALS AND METHODS

### Cells and viruses.

BHK-21 cells (baby hamster kidney cell line) and HeLa cells were cultured and maintained in Dulbecco’s modified Eagle’s medium (DMEM, Sigma) supplemented with 10% heat-inactivated fetal bovine serum (FBS), 100 U/ml penicillin, and 100 mg/ml streptomycin sulfate at 37°C in 5% CO_2_. WT JEV and NS1′-defective JEV viruses were generated as described previously. Briefly, WT JEV (SA14 strain, pSA14) and NS1′-defective JEV (rG66A, a G-to-A mutation at nucleotide position 66 in the NS2A gene of SA14) were produced by electroporation of BHK-21 cells with transcribed RNA from the full-length cDNA clones pMW218-JEV-rAT and pMW218-JEV-NS2A-G66A, respectively.

### Plasmid construction.

To construct the CDK1 and CDC25C expression plasmids, the corresponding coding regions from cDNA derived from HeLa cells were amplified by PCR and were cloned into the 3×FLAG-CMV-10 vector. The pcDNA3.1 plasmids encoding the JEV NS1, NS1′ which was constructed by inserting one nucleotide T before the heptanucleotide to abolish the expression of NS1, and ΔNS1′_52aa_ (the last 52 unique aa of NS1′ protein) were constructed as described previously (14). Primers used for PCR in this study are listed in Table 1. All constructs were verified by sequencing.

**TABLE 1:** Primers used in this study^*a*^

Primer	Sequence
JEV-NS1 and NS1′-F	CGGGATCCGACACTGGATGTGCCATTGACAT
JEV-NS1′_52aa_-F	CGGGATCCTTTAATGGTGAAATGGTTGAC
JEV-NS1-R	CCCTCGAGTCAAGCATCAACCTGTGATCTAACGA
JEV-NS1′-R	CCCTCGAGTCAGTGTAAGTGATGCCCCCAAGCAT
hCDK1-F	CCAAGCTTGCCACCATGGAAGATTATACCAAAATAGAGAAAATT
hCDK1-R	CGCGGATCCGCG TCA CATCTTCTTAATCTGATT
hCDC25C-F	CCAAGCTT GCCACC ATGTCTACGGAACTCTTCTCATC
hCDC25C-R	CGCGGATCC TCA TCATGGGCTCATGTCCTTCACC
qRT-PCR	
miR-22 stem-loop	GTCGTATCCAGTGCAGGGTCCGAGGTATTCGCACTGGATACGACACAGTT
miR-22-F	AAGCTGCCAGTTGAAGAACTGT
Universal reverse	GTGCAGGGTCCGAGGT
hIFN-β-F	TGCTCTGGCACAACAGGTAG
hIFN-β-R	AGCCTCCCATTCAATTGCCA
hβ-actin-F	AGCGGGAAATCGTGCGTGAC
hβ-actin-R	GGAAGGAAGGCTGGAAGAGTG
hMAVS-F	CCGTTTGCTGAAGACAAG
hMAVS-R	GCTCTGGTAGACAGAGGC
JEV-F	TGGTTTCATGACCTCGCTCTC
JEV-R	CCATGAGGAGTTCTCTGTTTCT
JEV-probe	FAM-CCTGGACGCCCCCTTCGAGCACAGCGT-TAMRA
RNA oligonucleotides	
miR-22 inhibitor control	ACAGUUCUUCAACUGGCAGCUU
Inhibitor	CAGUACUUUUGUGUAGUACAA

a h, human; F, forward; R, reverse.

### Reagents.

Primary antibodies to FLAG, GAPDH, CDK1, p-CDK1 (Thr-15), and p-CREB (Ser-133) were purchased from Abclonal Technology. Other antibodies used include anti-MAVS (Proteintech Technology), anti-CREB (Abcam), anti-c-Rel and anti-p-IκBα (S32/35) (Cell Signaling Technology), and anti-mouse/rabbit secondary antibodies labeled with horseradish peroxidase (Boster). The monoclonal antibodies against JEV NS5, NS1 (bind to both NS1 and NS1′), and NS1′ (specifically bind to NS1′) were generated in our laboratory (26). Poly(I·C) was obtained from Sigma. CDK1 inhibitor RO3306 was purchased from Selleck.

### Purification and identification of NS1′-interacting cellular proteins.

HeLa cells infected with the WT JEV at an MOI of 1.0 for a period of 36 h were subjected to lysis with RIPA buffer (Sigma) containing protease inhibitor cocktail (Roche). The cell lysate was incubated with the normal or JEV NS1′ antibodies at 4°C overnight. Protein A+G agarose beads (50 μl; Beyotime) were added and incubated for another 3 h. The agarose beads were subsequently washed three times with wash buffer (0.05 M Tris-HCl with 0.15 M NaCl). The purified products were separated by sodium dodecyl sulfate-polyacrylamide gel electrophoresis (SDS-PAGE) and visualized by silver staining. The stained bands were excised, digested in gels with Lys-C, and analyzed by the direct nanoflow liquid chromatography-tandem mass spectrometry (LC-MS/MS) system.

### Cell viability assay.

The viability of cultured cells was detected using the MTT cell proliferation and cytotoxicity assay kit (Beyotime) according to the manufacturer’s instruction. Briefly, HeLa cells were seeded in 96-well white opaque plates at a density of 5 × 10^4^ cells/ml. After incubation for 12 h at 37°C with 5% CO_2_, the culture supernatants were replaced with different concentrations of RO3306 or DMSO, followed by incubation for another 24 h. Subsequently, the cells were exposed to MTT solution (5 mg/ml) for 4 h, and then the formazan solvent was added for detection by a spectrophotometer at 570 nm. The quantification of luminescence signals under each condition was compared with its corresponding DMSO control.

### Virus binding and internalization assays.

HeLa cells were treated with RO3306 or DMSO prior to infection with WT JEV virus at an MOI of 10 at 4°C for 1 h, allowing the viruses to attach to the cell surface without entering. Later, the cells were washed with phosphate-buffered saline (PBS) to remove the unattached viral particles, and the amount of RNAs from viral particles that had attached to the cell surface was measured by quantitative real-time PCR (qRT-PCR). In a subsequent experiment, cells were further incubated at 37°C for 1 h to initiate viral entry, and then the infected cells were stringently washed with an alkaline high-salt solution to remove free virus as well as the cell surface-associated virus, and the intracellular viral RNAs were quantified using qRT-PCR.

### Co-IP.

HeLa cells were transfected with the plasmids indicated in the figures. At 36 h posttransfection, cell extracts were prepared using RIPA buffer (Sigma) containing protease inhibitor cocktail (Roche). The cell lysate was incubated with the indicated antibodies at 4°C overnight. Protein A+G agarose beads (50 μl; Beyotime) were added and incubated for another 3 h. The agarose beads were subsequently washed three times with wash buffer (0.05 M Tris-HCl with 0.15 M NaCl). The bound proteins were eluted by boiling in the SDS-PAGE loading buffer for 10 min and then used for Western blotting with the indicated antibodies.

### Western blotting.

Total cell lysates were generated with RIPA buffer (Sigma) containing proteinase and phosphatase inhibitors (Roche). Protein concentrations were measured using a bicinchoninic acid (BCA) protein assay kit (Thermo Scientific). Equal protein quantities were separated by SDS-PAGE (10% polyacrylamide), transferred to polyvinylidene difluoride membranes, incubated with primary and secondary antibodies, and visualized with an enhanced chemiluminescence reagent (Bio-Rad).

### RNA extraction and qRT-PCR.

Whole-cell RNA extraction was performed using the TRIzol reagent (Magen). Reverse transcription of RNAs was performed with a first-strand cDNA synthesis kit (Abclonal) according to the manufacturer’s instructions. The RNA transcripts corresponding to miR-22 were reverse transcribed using the miR-22-specific stem-loop primer. qRT-PCR was performed by using the SYBR green PCR master mix (Abclonal) and the 7500 real-time PCR system (Applied Biosystems). For MAVS and IFN-β expression, data were normalized to the level of β-actin in each sample. The expression of miR-22 was normalized to the level of U6 small nuclear RNA in each sample. Amplification was carried out successively as follows: 2 min at 50°C and 10 min at 95°C, followed by 40 cycles of 95°C for 15s, 60°C for 15s, and 72°C for 30s. Primers used are listed in Table 1.

### Plaque assay.

Virus loads in cell culture supernatants and mice brain tissues were assessed by plaque assay in BHK-21 cells. The supernatants of cell cultures were harvested and stored at −80°C. Samples were serially diluted and inoculated onto the monolayers of BHK-21 cells at 37°C for 1 h. Then, the supernatants were removed and cells were washed thrice with serum-free DMEM. The infected cells were further incubated for 4 days in DMEM containing 3% fetal bovine serum and 1.5% sodium carboxymethyl cellulose (Sigma). Finally, viral titers were calculated based on visible plaques after staining with crystal violet. All data are expressed as the mean of triplicate samples.

### ChIP assay.

Chromatin immunoprecipitation (ChIP) assay was performed according to the manufacturer’s protocols of the ChromaFlash high-sensitivity ChIP kit (EpiGenTek) with minor modifications. A total of 2 × 10^6^ HeLa cells were transfected with 3×FLAG-CMV-10-CDK1 or an empty vector. Treated cells were harvested at 36 hpi by cross-linking in a final concentration of 1% formaldehyde. Cross-linking was stopped after 5 min by adding glycine to a final concentration of 125 mM. The cells were then sonicated to a fragment size range of 100 to 700 bp. Immunoprecipitation was performed by incubating sheared chromatin overnight at 4°C with c-Rel antibody, CREB antibody, or nonimmunogenic IgG (rabbit antiserum). PCR products were resolved by 2% agarose ethidium bromide gel electrophoresis and visualized by UV light. The expression level of a target DNA sequence was determined relative to its abundance in the input chromatin and represented as fold enrichment compared with that of the uninfected control.

### Animal experiments.

MAVS^−/−^ mice (C57BL/6 × 129Sv/Ev) and their WT control mice (5 weeks old) were kindly provided by Ling Zhao (State Key Laboratory of Agricultural Microbiology, Huazhong Agricultural University, Wuhan, China). All animal experiments were carried out according to the National Institute of Health Guide for the Care and Use of Laboratory Animals, and the experimental protocols were approved by the Scientific Ethics Committee of Huazhong Agricultural University (HZAUMO-2019-040). WT or MAVS^−/−^ mice were randomly assigned to four groups: group 1, only DMSO-treated group (DMSO); group 2, only RO3306-treated group (RO3306); group 3, JEV-infected and DMSO-treated group (DMSO+JEV); and group 4, JEV-infected and RO3306-treated group (RO3306+JEV). Mice belonging to the DMSO+JEV and RO3306+JEV groups were injected intraperitoneally with 10^4^ PFU of the WT JEV P3 strain in 100 μl of DMEM, whereas mice in the other two groups received 100 μl of DMEM. On day 3 postinfection, RO3306 (10 mg/kg body weight) or the equal volume of DMSO was administered intravenously in mice. On days 3 and 4 postinfection, 5 mice from each group were sacrificed, and serum and brain samples were harvested for subsequent experiments. The remaining mice (*n* = 10 per group) were monitored daily to record clinical signs of the disease and mortality. Based on the corresponding reference, we divided clinical signs of the disease into seven grades: subclinical, ruffled fur and hunched, hindlimb weakness, mild paresis, moderate paresis, severe paresis, and moribund or dead (45).

### Statistical analysis.

All experiments were carried out at least three times. Analyses were conducted using Prism version 6.0 (GraphPad Software). Results are expressed as mean ± standard error of the mean (SEM). Data were compared either with a two-way analysis of variance (ANOVA) with subsequent tests using Bonferroni posttests for multiple comparisons or with the Student’s *t* test. The survival rate was analyzed via log-rank test. For all tests, *P* < 0.05 was considered to be significant.

### Data availability.

The mass spectrometry proteomics data have been deposited to the ProteomeXchange Consortium via the PRIDE (46) partner repository with the data set identifier PXD027412 (username: reviewer_pxd027412@ebi.ac.uk, password: NDc5vcFA).
