# Supplementary material for: Japanese Encephalitis Virus NS1′ Protein Interacts with Host CDK1 Protein to Regulate Antiviral Response
Source: Microbiol Spectr. 2021 Nov 10;9(3):e01661-21. doi: 10.1128/Spectrum.01661-21 (PMC8579942; doi:10.1128/Spectrum.01661-21)
Supplement: SUPPLEMENTAL FILE 1 — Supplemental material. Download SPECTRUM01661-21_Supp_1_seq11.pdf, PDF file, 1.0 MB [file spectrum01661-21_supp_1_seq11.pdf]

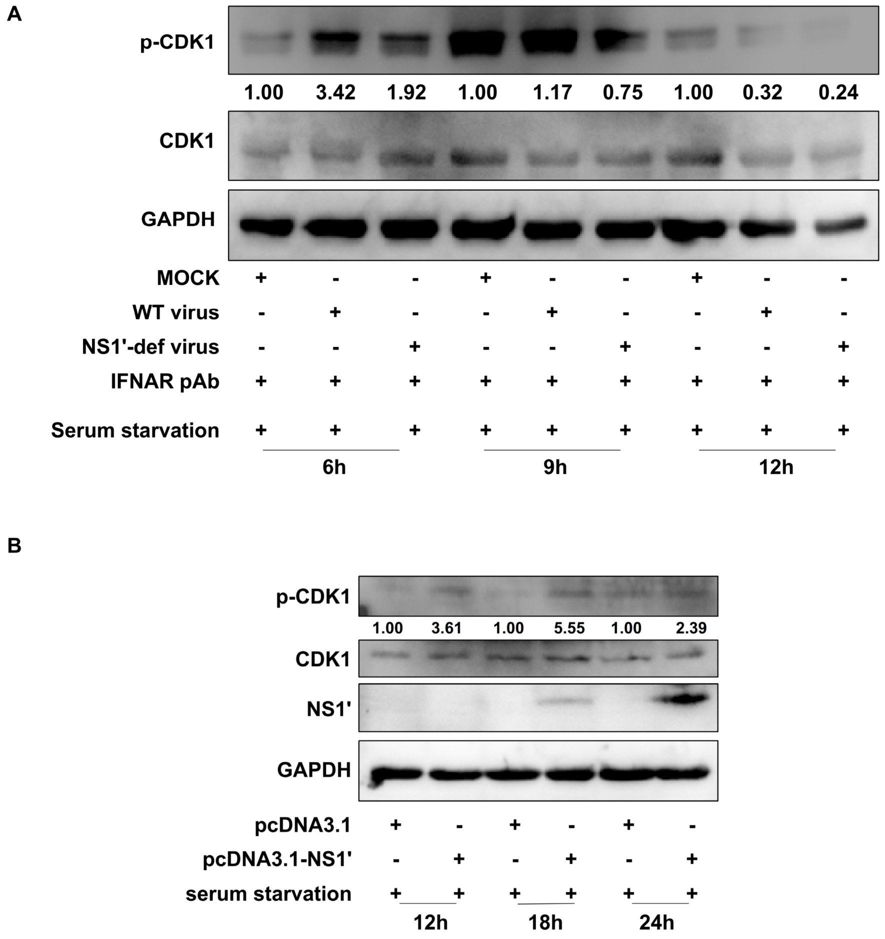

Supplementary Fig 1. JEV NS1' enhances the CDK1 phosphorylation after the cell cycle being synchronized. (A) HeLa cells were serum starved for 24h, and then infected with WT virus or NS1'-def virus at an MOI of 1.0. The cells were harvested at 6, 9, and 12 hpi. Whole-cell protein lysates were separated by SDS-PAGE and examined by Western blot analysis using indicated antibodies. (B) HeLa cells were serum starved for 24h, and then transfected with pcDNA3.1-NS1' or empty vector. After 12, 18, and 24h, cell lysates were subjected to Western blotting using indicated antibodies.

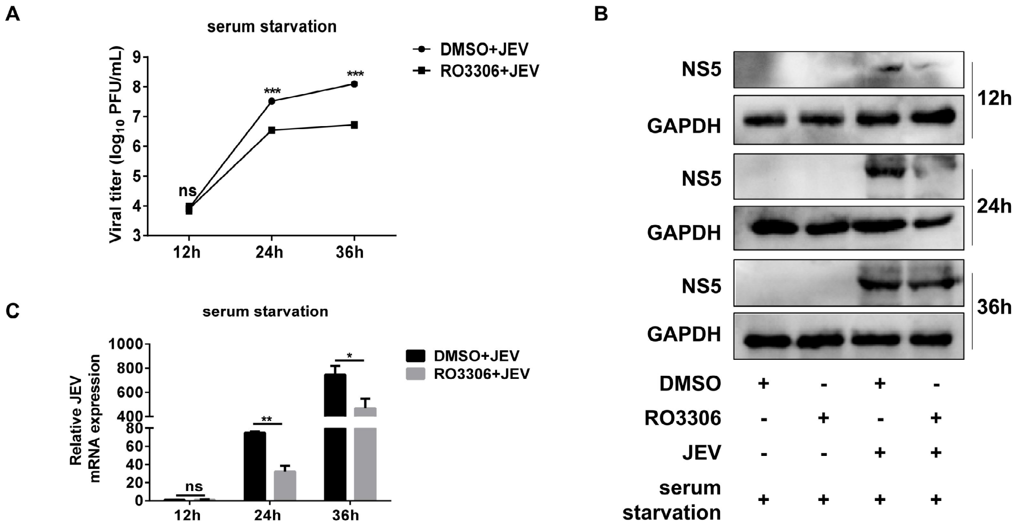

Supplementary Fig 2. CDK1 positively regulates the replication of JEV at the cell cycle being synchronized. (A-C) HeLa cells were serum starved for 24h, followed by infection with WT virus or NS1'-def virus at an MOI of 1.0, and then the cells were harvested at 6, 9, and 12 hpi. Viral titers (A), JEV NS5 protein expression (B), and total viral RNA expression (C) were measured by plaque assay, Western blot, and qRT-PCR, respectively. *p < 0.05, **p < 0.01, ***p < 0.001. ns, non-significant.
